# Integrated Transcriptomic and Metabolomic Profiling Reveals Monotonic Molecular Signatures During Fruiting Body Development of *Coprinus comatus*

**DOI:** 10.3390/jof11120849

**Published:** 2025-11-29

**Authors:** Zhu Liu, Linzhi Kang, Yangyang Peng, Jianhao Wang, Luyao Ye, Hui Zhou, Ming Liu

**Affiliations:** 1 Guangdong Provincial Key Laboratory of Utilization and Conservation of Food and Medicinal Resources in Northern Region, Shaoguan University, Shaoguan 512005, China; liuzhu@sgu.edu.cn; 2 Guangdong Key Laboratory for New Technology Research of Vegetable, Vegetable Research Institute, Guangdong Academy of Agricultural Sciences, Guangzhou 510640, China; pengyangyang@gdaas.cn (Y.P.); zhouhui@gdaas.cn (H.Z.); 3 School of Biology and Agriculture, Shaoguan University, Shaoguan 512005, China; 4 School of Food, Shaoguan University, Shaoguan 512005, China; lzkang@sgu.edu.cn

**Keywords:** *Coprinus comatus*, fruiting-body development, transcriptomics, metabolomics, differential expression

## Abstract

Integrative transcriptomic–metabolomic profiling across three developmental stages of *Coprinus comatus* revealed that a coherent, monotonic molecular program drives fruiting body maturation. Stage-resolved RNA-seq and untargeted metabolomics were carried out to cleanly separate samples, and results showed directional trends: glycerophospholipids and other lipid species declined steadily, whereas peptides and amino-acid derivatives accumulated. Early development was driven by cell cycle regulation and DNA damage repair, while later stages shifted toward amino acid, glutathione and cell wall metabolism. These coupled transcriptional and metabolic shifts delineate processes associated with maturation and autolysis from which actionable markers can be identified to refine cultivation practices and optimize harvesting time.

## 1. Introduction

*Coprinus comatus* (O.F. Müll.) Pers., commonly known as the shaggy mane or “Jituigu” in China, is an edible basidiomycete prized for its medicinal and nutritional value [1]. This mushroom has been traditionally used as a functional food due to its various health-promoting properties, including anti-hyperglycemic (blood-glucose-lowering), hypolipidemic (cholesterol-lowering), anti-tumor, and antioxidant effects [2]. Biochemical studies have identified multiple bioactive constituents in *C. comatus*. These include polysaccharides from liquid-cultured mycelia, which exhibit significant antioxidant and glucose uptake activity [1]; fruiting-body extracts, which modulate adipogenesis via PPARγ/Akt signaling [1]; and a protein (laccase) which, when isolated from the mushroom, shows potent anti-proliferative effects on cancer cells [1]. Owing to its rich nutraceutical profile, *C. comatus* is now widely cultivated in Asia and marketed as a medicinal mushroom. In China alone, it has become an important commodity crop, with annual production exceeding 1.4 × 10^8^ kg [1]. However, a major challenge in *C. comatus* cultivation is understanding this species’ ephemeral fruiting body—as they mature, they rapidly undergo autolysis, a self-digesting process that causes the cap to liquefy into “ink,” drastically reducing shelf life and harvest yields [3]. This propensity for deterioration post-maturity necessitates a deeper understanding of the mushroom’s developmental biology so that cultivation strategies can balance maximizing the content of bioactive compounds with minimizing losses from autolysis.

Advances in high-throughput omics have resulted in powerful tools capable of dissecting the molecular changes underpinning mushroom development. Transcriptomic (RNA-Seq) analyses have been used in various Basidiomycetes to identify genes regulating fruiting body formation and maturation [1]. For instance, studies in *Pleurotus* and *Morchella* revealed stage-specific gene expression patterns associated with primordia initiation and vegetative growth, respectively [4]. Until recently, however, such genome-wide expression data were lacking for *C. comatus* [1]. Likewise, metabolomic profiling can illuminate the biochemical pathways active at different stages, complementing transcript data [5]. Integrating transcriptomics and metabolomics is particularly valuable for mushrooms, which often reconfigure their metabolism during development—as spores mature, nutrients are reallocated, secondary metabolites are synthesized, and cellular components degrade [1]. By correlating gene expression with metabolite levels, pathways can be identified that are either up- or downregulated as the fungus progresses from juvenile to mature stages. Such insight is directly relevant to cultivation: knowing when key nutrients or bioactive compounds accumulate can inform harvest times or interventions to enhance target compounds [6].

In this study, we performed a comprehensive multi-omics analysis of *C. comatus* fruiting bodies across three developmental stages: juvenile (JTGX: young, small-cap stage), intermediate (JTGZ: mid-sized stage), and mature (JTGD: fully developed pre-autolysis stage). High-throughput RNA sequencing (transcriptome) and untargeted metabolite profiling (metabolome) were applied to each stage, yielding a dynamic overview of changes to gene expression and metabolite abundance during mushroom development.

## 2. Materials and Methods

### 2.1. Sample Collection and Preparation

The *Coprinus comatus* strain was preserved in the Cultivated Edible Fungi Germplasm Resource Bank of Guangdong Province, Guangdong Key Laboratory for New Technology Research of Vegetables. *Coprinus comatus* was cultivated on mixed substrates including 70% wood chips, 20% cottonseed hulls, and 10% wheat bran with 65% water content.

Fruiting bodies of *C. comatus* were collected at three distinct developmental periods: juvenile (JTGX), intermediate (JTGZ), and mature (JTGD) stages. Each group contained six biological replicates for a total of 18 samples. All specimens were immediately frozen in liquid nitrogen and stored at –80 °C until subsequent metabolomic and transcriptomic analyses were performed.

### 2.2. De Novo Assembly and Annotation

Genomic DNA extraction and long-read sequencing. High-molecular-weight DNA was isolated from *C. comatus* tissue and subjected to Oxford Nanopore long-read sequencing following standard library preparation and base-calling workflows. Sequencing outputs were quality-checked and filtered prior to assembly [7,8].

De novo assembly and polishing. Filtered long reads were assembled de novo. Draft contigs were iteratively polished using long-read signal-based correctors, and short-read evidence was used when available to reduce indels and improve the accuracy of the consensus. Assembly statistics and integrity were evaluated using common metrics (e.g., N50) and BUSCO single-copy ortholog recovery [9,10,11,12].

Repeat masking and gene prediction. Repeats were identified and soft-masked prior to constructing a gene model. Ab initio predictors and evidence-guided approaches were combined to generate protein-coding gene models, followed by the identification of non-coding RNAs [13,14].

**Figure 1:**
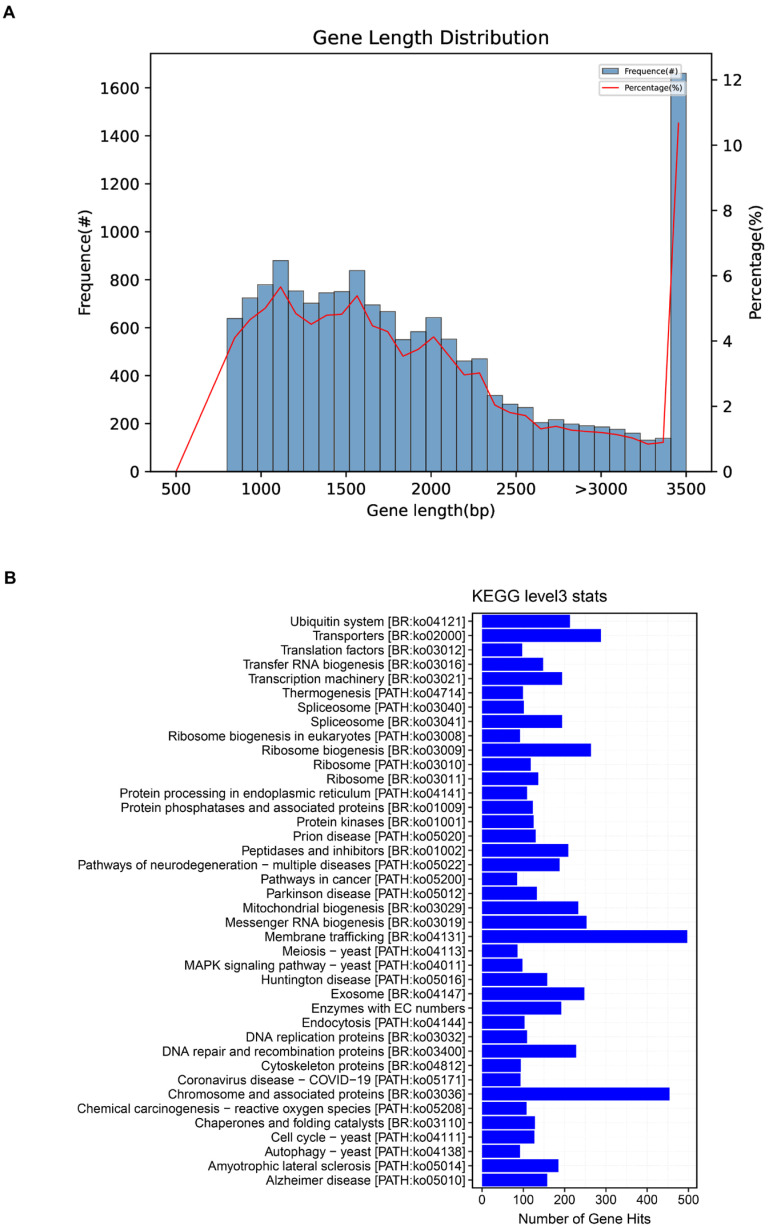
Genomic features of *Coprinus comatus*. Length distribution of coding sequences (CDSs) predicted from the assembled genome. Only CDSs ≥ 90 bp were included; bars indicate the number of genes in each length interval, while the line represents their proportion relative to the total gene set. The majority of CDSs cluster in the 1–2 kb range, consistent with an average length of ~1.55 kb (**A**). KEGG Level-3 distribution of KO assignments derived from the *C. comatus* genome. Bars indicate the number of genes annotated to each pathway; prominent categories include ribosome, spliceosome, and protein processing in endoplasmic reticulum, together with broad metabolism (amino acids and carbohydrates) and transporter modules. Note that these counts report annotation coverage rather than differential or enriched activity; functional enrichment is assessed separately using stage-specific DEGs/DAMs (see Results Section 3.3 and Section 3.5). Source data: KO mapping files produced during genome annotation (**B**).

Functional annotation. Predicted proteins were functionally annotated against multiple resources (NR/Swiss-Prot/Pfam/GO/KEGG). KEGG Orthology (KO) identifiers were assigned and summarized across pathway levels, producing per-gene and per-pathway tables (e.g., gene2ko.txt, ko_sum.xls, Level*.xls) and pathway map highlights (Map/). These KO-level resources relate to the KEGG Level-3 census presented in Figure 1.

### 2.3. Metabolomic Analysis

#### 2.3.1. LC–MS Data Acquisition

LC-MS/MS analyses were performed using a UHPLC system (Vanquish, Thermo Fisher Scientific, Waltham, MA, USA) with a Waters ACQUITY UPLC BEH Amide (2.1 mm × 50 mm, 1.7 μm) coupled to an Orbitrap Exploris 120 mass spectrometer (Orbitrap MS, Thermo Fisher Scientific, Waltham, MA, USA). The mobile phase consisted of 25 mmol/L ammonium acetate and 25 mmol/L ammonia hydroxide in water (pH = 9.75) (A) and acetonitrile (B). The auto-sampler temperature was 4 °C, and the injection volume was 2 μL. The Orbitrap Exploris 120 mass spectrometer was used due to its ability to acquire MS/MS spectra using the information-dependent acquisition (IDA) mode controlled by acquisition software (Xcalibur Version 4.4, Thermo Fisher Scientific, Waltham, MA, USA). In this mode, the acquisition software continuously evaluates the full-scan MS spectrum. ESI source conditions were set as follows: sheath gas flow rate at 50 Arb, Aux gas flow rate at 15 Arb, capillary temperature of 320 °C, full MS resolution at 60,000, MS/MS resolution at 15,000, and collision energy of SNCE 20/30/40, and spray voltage at 3.8 kV (positive) or −3.4 kV (negative), respectively. Quality control (QC) samples were injected periodically to ensure instruments were stable.

#### 2.3.2. Data Preprocessing

Raw LC–MS data were processed using peak detection, deconvolution, and alignment. Peaks were retained if missing values did not exceed 50% within any group, and missing values were imputed using half of the minimum observed value. Peak intensities were normalized to internal standards. Metabolite annotation was performed using the Human Metabolome Database (HMDB), KEGG (Kyoto Encyclopedia of Genes and Genomes), and PubChem databases [15].

### 2.4. Transcriptomic Analysis

#### 2.4.1. RNA Extraction, Library Preparation, and Sequencing

Total RNA was isolated from fruiting body tissues using a TRIzol-based method. RNA quality and concentration were assessed using an Agilent Bioanalyzer(Agilent Technologies, Santa Clara, CA, USA), and Poly(A)-mRNA was enriched with Oligo(dT) magnetic beads, fragmented, and reverse-transcribed into cDNA. Following end repair, A-tailing, and adapter ligation, cDNA libraries were amplified by PCR. Sequencing was performed on the Illumina HiSeq 2500 platform(Illumina, San Diego, CA, USA).

#### 2.4.2. Quality Control and Alignment

Raw reads were processed with fastp to remove adapters and low-quality bases. Clean reads were aligned to the reference genome using Bowtie2, and transcript assembly was carried out with StringTie Version 2.2.6 [7,16,17,18].

#### 2.4.3. Expression Quantification and Differential Analysis

Transcript abundances were quantified using Salmon, and gene-level counts were summarized with tximport. Normalized expression values (TPM) and raw counts were obtained for downstream analysis. Differentially expressed genes (DEGs) were identified using DESeq2 Version 1.34.0, applying adjusted thresholds of *p* < 0.05 and |log_2_ fold change| ≥ 1. Functional annotation and KEGG pathway enrichment were subsequently conducted to interpret biological significance [19].

### 2.5. Statistical Analysis

Multivariate and univariate statistical methods were used to identify stage-associated changes at both metabolite and gene levels. Principal component analysis (PCA) and orthogonal partial least squares–discriminant analysis (OPLS–DA) were applied to LC–MS metabolomics data to identify global metabolic differences among developmental stages [20,21,22]. At the univariate level, Student’s *t*-tests were used to compare metabolite intensities between stages; the resulting *p*-values were further adjusted for multiple testing using the Benjamini–Hochberg procedure, and metabolites with FDR ≤ 0.05, |log_2_ fold change| ≥ 1 and variable importance in projection (VIP) ≥ 1 were defined as differentially abundant metabolites (DAMs) [23]. For the RNA-seq data, transcript abundances were quantified with Salmon and summarized to gene-level counts using tximport. Differentially expressed genes (DEGs) were identified using DESeq2 with Benjamini–Hochberg-adjusted correction *p* < 0.05 and |log_2_ fold change| ≥ 1. DAMs and DEGs were subsequently subjected to KEGG pathway annotation and KEGG over-representation analysis to interpret changed biological processes (significance threshold *p* < 0.05).

### 2.6. qRT-PCR Validation of Differentially Expressed Genes

To validate the accuracy of the RNA-seq results, six differentially expressed genes predicted to encode glutathione S-transferases (GSTs) were selected for quantitative real-time PCR (qRT-PCR) analysis. Total RNA was extracted from JTGX, JTGZ, and JTGD tissues using TRIzol reagent (Invitrogen, Carlsbad, CA, USA) following the manufacturer’s protocol. For each sample, 2 μg of total RNA was reverse-transcribed into cDNA using the Prime-Script™ RT Reagent Kit (Takara, Tokyo, Japan).

qRT-PCR was performed on an Applied Biosystems QuantStudio™ 5 Real-Time PCR System (Thermo Fisher Scientific, Waltham, MA, USA) using TB Green^®^ Premix Ex Taq™ II (Takara, Tokyo, Japan). Actin2 was selected as the internal reference gene after confirming its stable expression across the three developmental stages. Three biological replicates and four technical replicates were included for each sample. Statistical significance was assessed using one-way ANOVA, with *p* < 0.05 considered significant. Primer sequences used for qRT-PCR are listed in Supplementary Table S1.

## 3. Results

### 3.1. Whole-Genome Sequencing, Assembly, and Functional Annotation

The assembled genome of *Coprinus comatus* was generated using Oxford Nanopore long-read sequencing and assembled de novo using Canu (v2.2), with subsequent polishing performed using Pilon and scaffolding via SSPACE and BLASR. The final assembly yielded 114 contigs with a total genome size of approximately 46.02 Mb and a GC content of 42.54%. The N50 value of the assembly reached 998,269 bp, indicating high continuity and quality, and the largest contig assembled was approximately 2.42 Mb. Assessment of genome completeness using the BUSCO (Benchmarking Universal Single-Copy Orthologs) pipeline showed high coverage, with 741 complete BUSCOs, including 605 single-copy and 136 duplicated orthologs out of 758 searched groups, indicating a high level of genome completeness and accurate assembly.

For the distribution of coding sequence (CDS) lengths in the *Coprinus comatus* genome, we only considered effective CDSs longer than 90 bp (Figure 1A). The *x*-axis represents the CDS length, while the bars denote the number of genes within each length interval, and the line indicates their percentage relative to the total gene set. The majority of predicted genes fall within the 1–2 kb range, consistent with the calculated average CDS length of ~1.55 kb (of the 15,555 predicted genes included, 15,429 were complete with both start and stop codons). By contrast, very short (<500 bp) and very long (>4 kb) CDSs occur only at low frequencies, indicating that the gene set is dominated by medium-length coding regions. This “enrichment in the mid-length range with tapering at both extremes” represents a typical feature of eukaryotic, particularly fungal, genomes and further supports the accuracy of the assembly and annotation as corroborated by GeneMark predictions and BUSCO completeness assessments. In addition to protein-coding genes, a total of 363 tRNA genes and 44 rRNA genes were predicted through tRNAscan-SE Version 2.0.10 and Barrnap Version 0.9 software, respectively.

To obtain a systems-level view of biochemical capacities in *Coprinus comatus*, we annotated the assembled genome against KEGG and summarized KO assignments at Level-3. The distribution was dominated by core information-processing and protein homeostasis modules, with ribosome, spliceosome, and protein processing in the endoplasmic reticulum exhibiting the highest gene counts. Substantial representation was also observed across amino-acid metabolism, carbohydrate metabolism, and transport categories; however, several disease-labeled KEGG maps (e.g., cancer and neurodegeneration collections) identified nontrivial findings that reflected conserved eukaryotic housekeeping pathways rather than evidence of pathology. Collectively, this map-level census indicates a gene repertoire primed for intensive translation, RNA processing, membrane trafficking, and redox/protein quality control—features that align with our stage-resolved transcriptome and metabolome trends during fruiting body growth and maturation (Figure 1B). Importantly, these bar lengths reflect an abundance of annotations (number of genes per pathway) but do not by themselves imply statistical enrichment. We evaluated this using stage-specific DEG/DAM sets in downstream analyses.

### 3.2. Global Metabolome Separation and Differential Features

**Figure 2:**
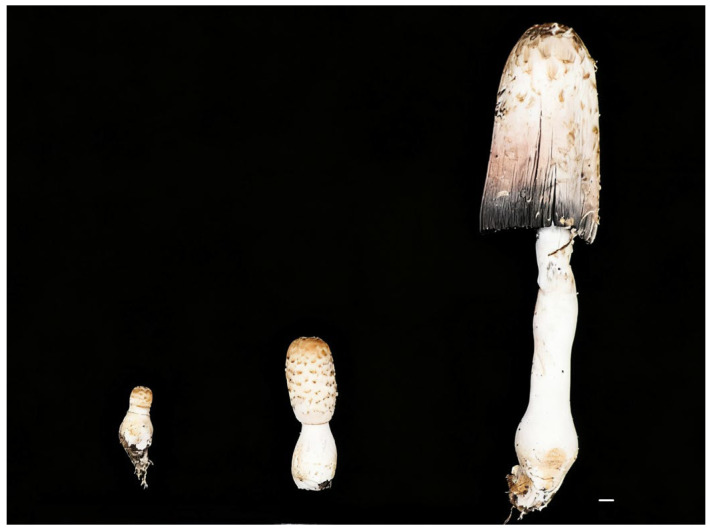
Morphometric comparison of *Coprinus comatus* fruiting bodies across three developmental stages (juvenile, mid, and mature periods; top to bottom). Total length increases from <5 cm to ~25–30 cm (~6–8×); cap diameter increases from ~1–2 cm to ~7–10 cm; and marked elongation and thickening of the stripe; the mature cap shows incipient deliquescence with inky coloration. Scale bar = 1 cm.

Fruiting bodies were stratified by morphology into juvenile (JTGX), intermediate (JTGZ), and mature stages (JTGD) (Figure 2). The same specimens underwent untargeted LC–MS metabolomics and RNA-seq with DAM/DEG calling, GO/KEGG enrichment, and transcript–metabolite integration.

**Figure 3:**
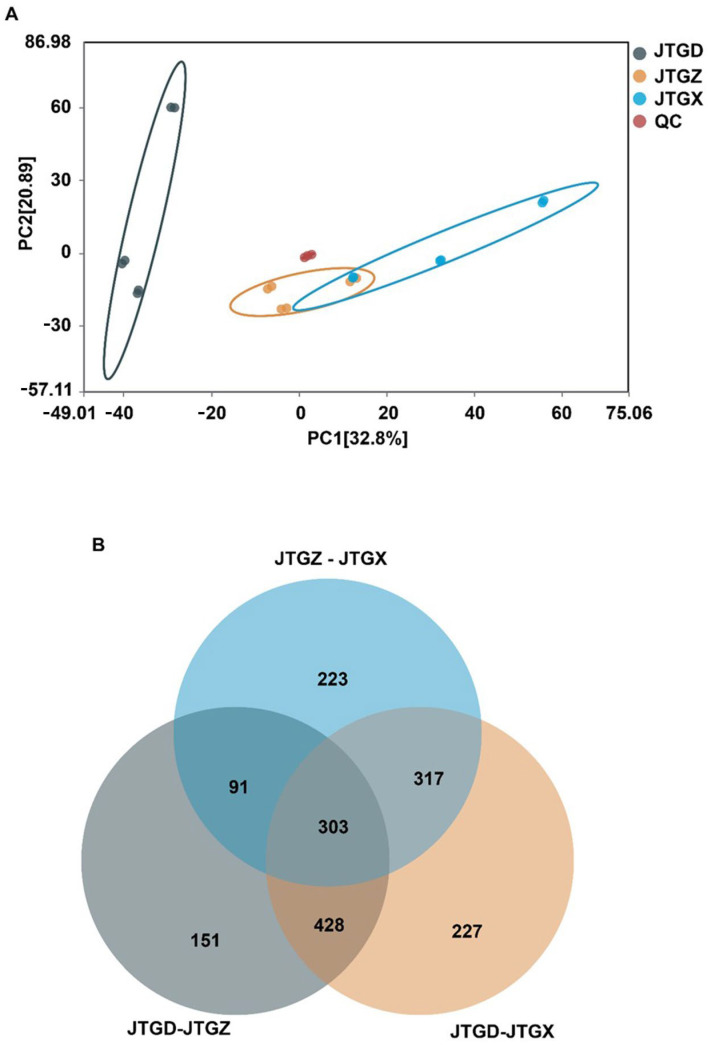
Global metabolomic variation across *Coprinus comatus* development stages. (**A**) PCA score plot of LC–MS metabolomic features showing clear separation of juvenile (JGTX), mid (JTGZ), and mature (JTGD) fruiting bodies (points are colored by stage; QC injections cluster tightly when present), indicating a dominant effect of stage on the metabolome. (**B**) Venn diagram summarizing differentially abundant metabolites identified in the three pairwise comparisons (JTGZ vs. JGTX, JTGD vs. JTGZ, and JTGD vs. JGTX under *p*-value significance and fold-change thresholds (*p* < 0.05; |log_2_FC| ≥ 1) (*n* = 6). Note: Positive log_2_FC is defined as having higher abundance in the second group of each contrast. The circle for JTGZ—JTGX is light blue; the circle for JTGD—JTGZ is gray; and the circle for JTGD—JTGX is light orange.

Untargeted LC–MS profiles of *Coprinus comatus* segregated into juvenile (JGTX), mid-stage (JGTZ), and mature (JTGD) samples were positioned into three non-overlapping clusters, with tight QC grouping. A dominant effect on the metabolome was identified for each stage (Figure 3A). Differentially abundant metabolites (DAMs; *p* < 0.05 and |log2FC| ≥ 1) from the three pairwise contrasts showed a shared core and stage-specific subsets, with contrasts involving JTGD contributing the largest unique fractions (Figure 3B).

### 3.3. Differential Metabolite Magnitudes and Directionality

**Figure 4:**
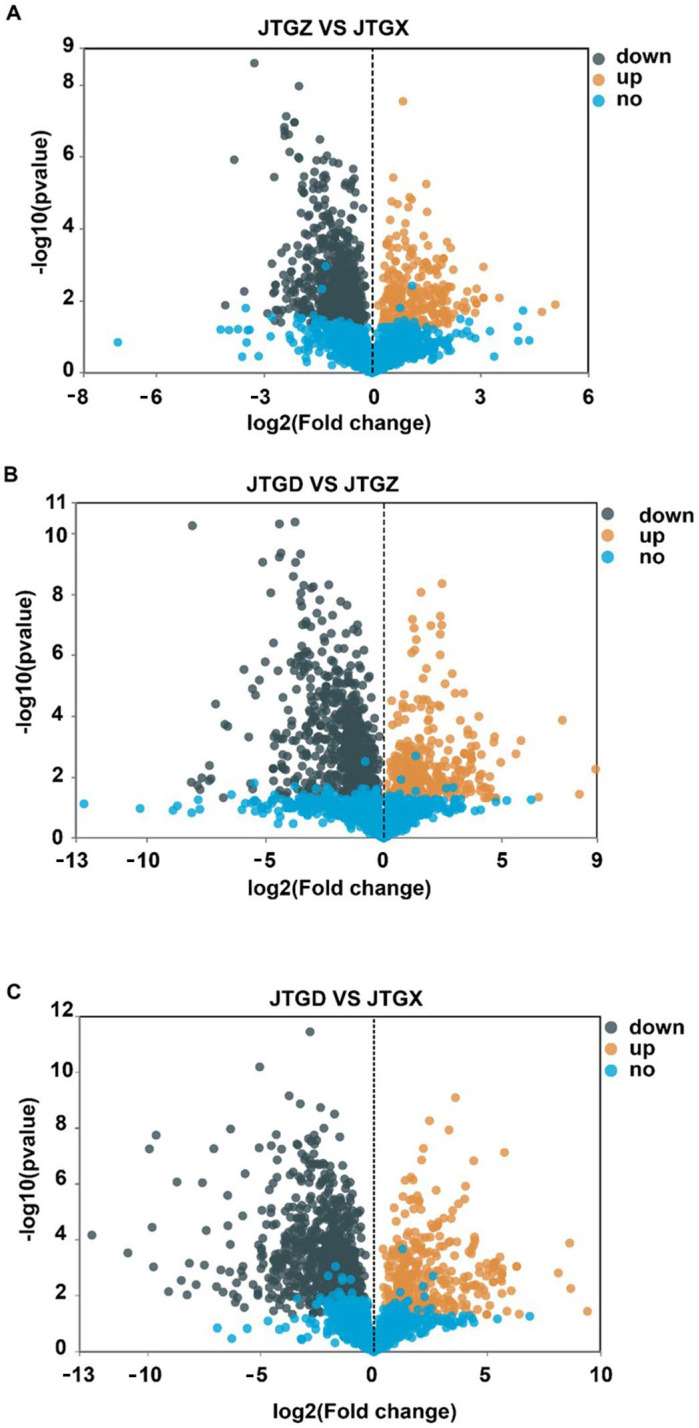
Volcano plots of differentially abundant metabolites across development comparisons of *Coprinus comatus*. (**A**) JTGZ vs. JGTX; (**B**) JTGD vs. JTGZ; and (**C**) JTGD vs. JGTX. Each plot displays a log_2_ fold change on the *x*-axis and −log_10_(*p*-value) on the *y*-axis; vertical dashed lines mark |log_2_FC| = 1 and the horizontal dashed line marks FDR = 0.05 (Benjamini–Hochberg correction). Metabolites beyond both thresholds are differentially abundant (DAMs) and colored by direction (the color scheme is indicated in the legend; by convention, positive log_2_FC denotes higher abundance in the second group of each contrast). Where shown, labels indicate the top-ranking DAMs by effect size or significance.

Volcano plots revealed numerous significant DAMs for each contrast with both positive and negative effect sizes beyond multiple-testing and fold-change thresholds (Figure 4A–C). The endpoint comparison for JTGD vs. JGTX displayed the broadest dispersion and the greatest density of significant features, indicating that it had the largest overall metabolomic shift across stages (Figure 4C).

### 3.4. Pathway Enrichment of Differential Metabolites

**Figure 5:**
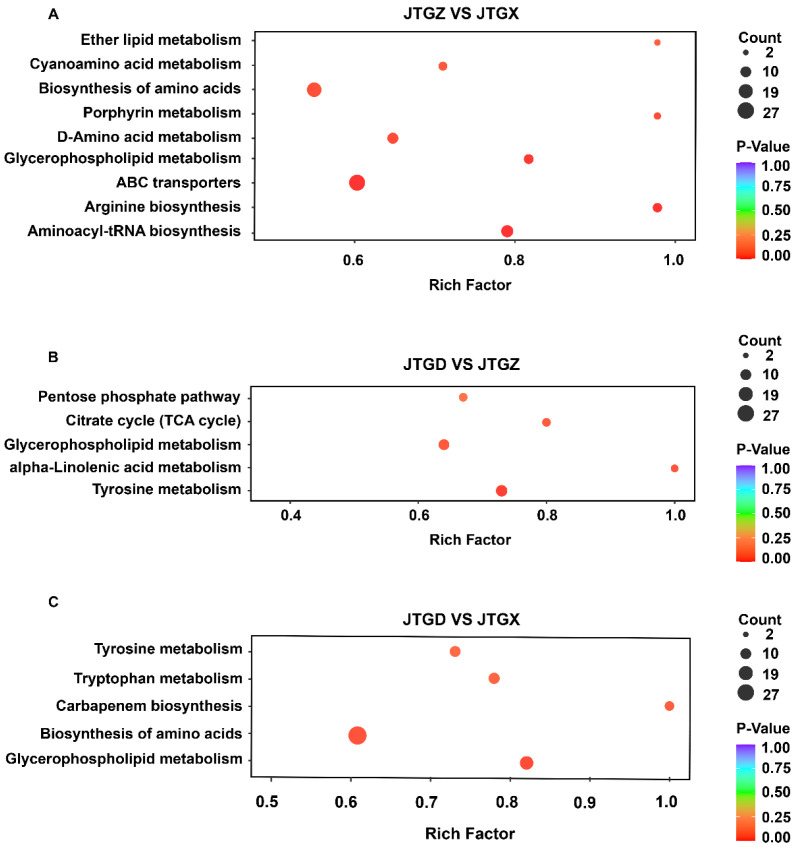
KEGG pathway enrichment of differential metabolites across comparisons of developmental stages in *Coprinus comatus*. Significantly different abundant metabolites (DAMs; *p* < 0.05; |log_2_FC| ≥ 1) from each pairwise contrast were subjected to KEGG over-representation analysis. (**A**) JTGZ vs. JGTX: ether lipid metabolism; cyanoamino-acid metabolism; biosynthesis of amino acids; porphyrin metabolism; D-amino-acid metabolism; glycerophospholipid metabolism; ABC transporters; arginine biosynthesis; and aminoacyl-tRNA biosynthesis. (**B**) JTGD vs. JTGZ: pentose phosphate pathway; citrate cycle (TCA cycle); glycerophospholipid metabolism; α-linolenic acid metabolism; and tyrosine metabolism. (**C**) JTGD vs. JGTX: tyrosine metabolism; tryptophan metabolism; carbapenem biosynthesis; biosynthesis of amino acids; and glycerophospholipid metabolism.

Stage-resolved enrichment analysis revealed distinct but partially overlapping pathway sets: In JTGZ vs. JGTX, we observed significant enrichment of ether lipid metabolism, cyanoamino-acid metabolism, biosynthesis of amino acids, porphyrin metabolism, D-amino-acid metabolism, glycerophospholipid metabolism, ABC transporters, arginine biosynthesis, and aminoacyl-tRNA biosynthesis (Figure 5A). In JTGD vs. JTGZ, the enriched terms included the pentose phosphate pathway, citrate cycle (TCA cycle), glycerophospholipid metabolism, alpha-linolenic acid metabolism, and tyrosine metabolism (Figure 3B). Finally, for the endpoint JTGD vs. JGTX contrast, we found tyrosine metabolism, tryptophan metabolism, carbapenem biosynthesis, biosynthesis of amino acids, and glycerophospholipid metabolism (Figure 5C). Across contrasts, glycerophospholipid metabolism was common to all three comparisons; biosynthesis of amino acids overlapped between JTGZ vs. JGTX and JTGD vs. JGTX, and tyrosine metabolism overlapped between JTGD vs. JTGZ and JTGD vs. JGTX. In contrast, ether lipid metabolism, cyanoamino-acid metabolism, porphyrin metabolism, D-amino-acid metabolism, ABC transporters, arginine biosynthesis, and aminoacyl-tRNA biosynthesis were unique to JTGZ vs. JGTX; the pentose phosphate pathway, Citrate cycle (TCA cycle), and alpha-linolenic acid metabolism were unique to JTGD vs. JTGZ; and tryptophan metabolism and carbapenem biosynthesis were unique to JTGD vs. JGTX (Figure 5A–C).

### 3.5. Global Transcriptome Separation and DEG Overlap

**Figure 6:**
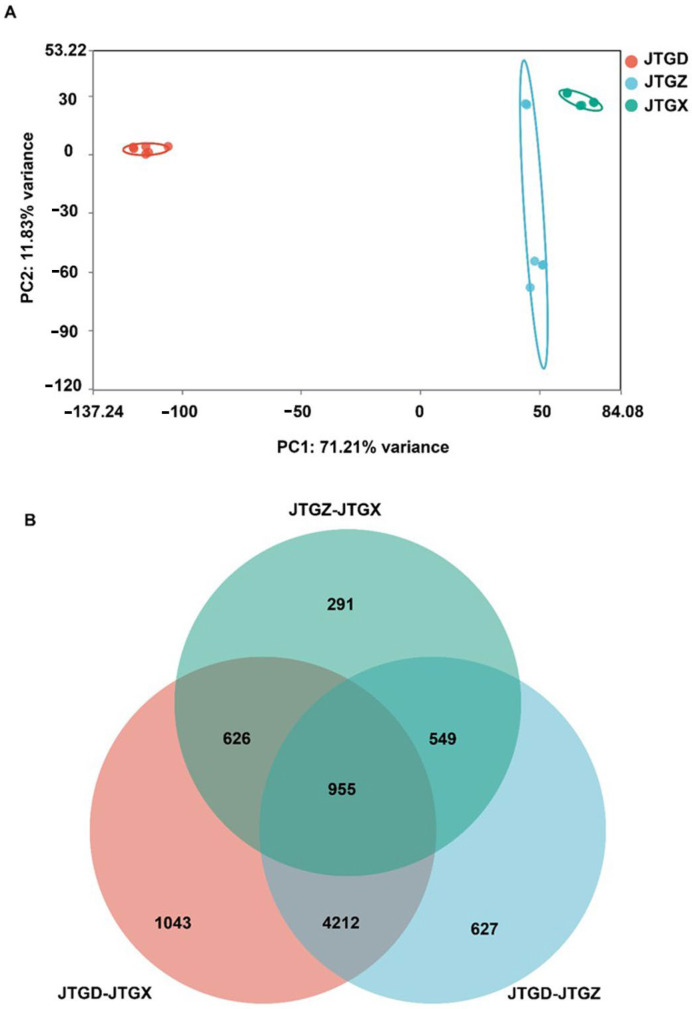
Global transcriptomic variation across *Coprinus comatus* development stages. (**A**) PCA score plot of RNA-seq expression profiles (variance-stabilized/normalized counts) showing clear separation of juvenile (JGTX), mid (JGTZ), and mature (JTGD) fruiting bodies, indicating stage-resolved transcriptomic programs. (**B**) Venn diagram summarizing differentially expressed genes (DEGs) identified in the three pairwise comparisons (JTGZ vs. JGTX, JTGD vs. JTGZ, and JTGD vs. JGTX under multiple-testing and effect-size criteria (*p*< 0.05; |log_2_FC| ≥ 1). Note: By convention, positive log_2_FC denotes higher expression in the second group of each contrast. The circle representing JTGD-JTGX is pink; the circle representing JTGZ-JTGX is mint green; and the circle representing JTGD-JTGZ is light blue.

RNA-seq profiles formed three well-separated clusters corresponding to JGTX, JGTZ, and JTGD, which indicated stage-resolved transcriptional programs (Figure 6A). DEG overlaps (*p* < 0.05; |log2FC| ≥ 1) showed a substantial intersection across contrasts alongside contrast-specific components; comparisons involving JTGD contributed to the majority of unique DEGs (Figure 6B).

### 3.6. Transcript-Level Effect Sizes and Counts

**Figure 7:**
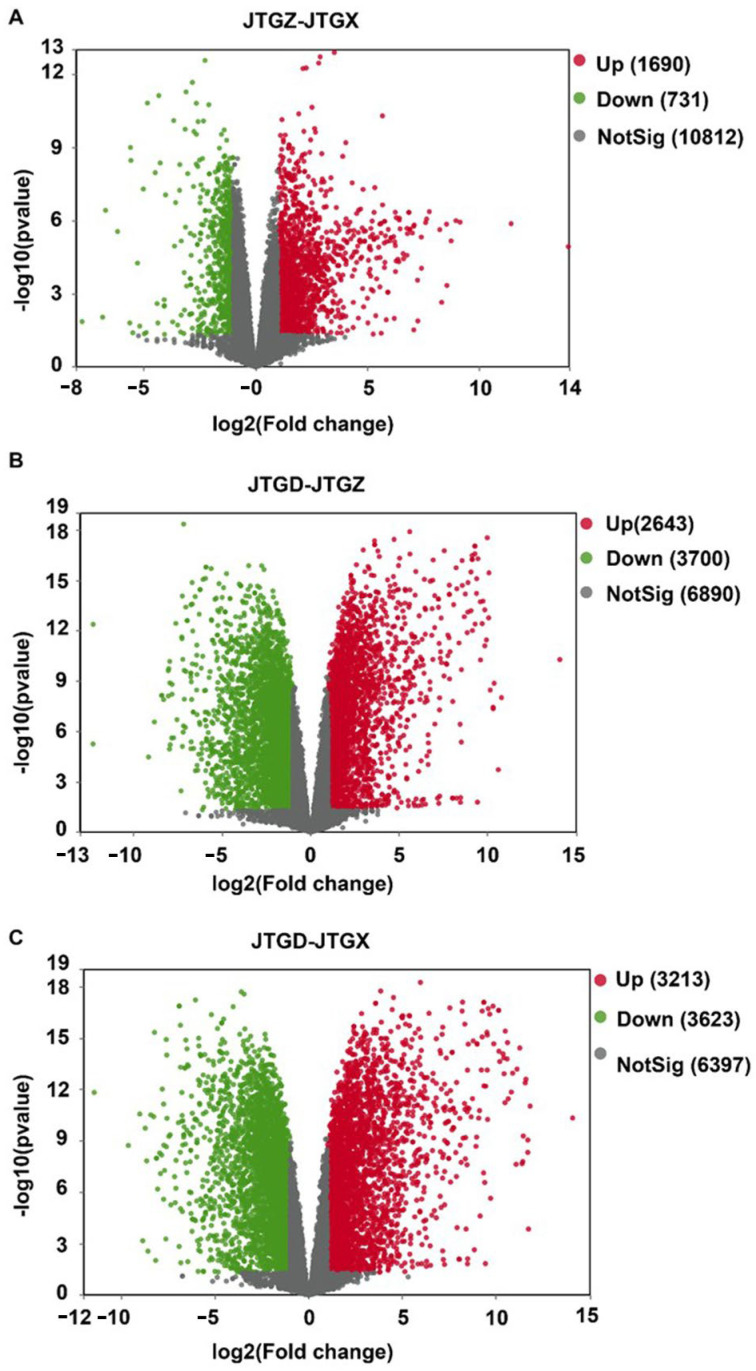
Volcano plots of differentially expressed genes across comparisons of *Coprinus comatus* developmental stages. (**A**) JTGZ vs. JGTX; (**B**) JTGD vs. JTGZ; and (**C**) JTGD vs. JGTX. Each panel displays log_2_ fold change (*x*-axis) versus −log_10_(*p*-value) (*y*-axis); vertical dashed lines mark |log_2_FC| = 1 and the horizontal dashed line marks *p* = 0.05 (Benjamini–Hochberg correction). Genes surpassing both thresholds are called differentially expressed (DEGs) and are colored by direction (legend indicates up/down); for convention, positive log_2_FC denotes higher expression in the second group of each contrast. Where shown, labels annotate representative top-ranking DEGs by effect size or significance.

Volcano plots summarized broad bidirectional transcriptional responses across all contrasts. Comparatively modest changes were observed from JGTX to JGTZ; marked reprogramming was observed from JGTZ to JTGD; and the most extensive dispersion and counts were identified at the endpoint JTGD vs. JGTX. This is consistent with the cumulative effect observed across development stages (Figure 7A–C).

### 3.7. Pathway Enrichment of DEGs

**Figure 8:**
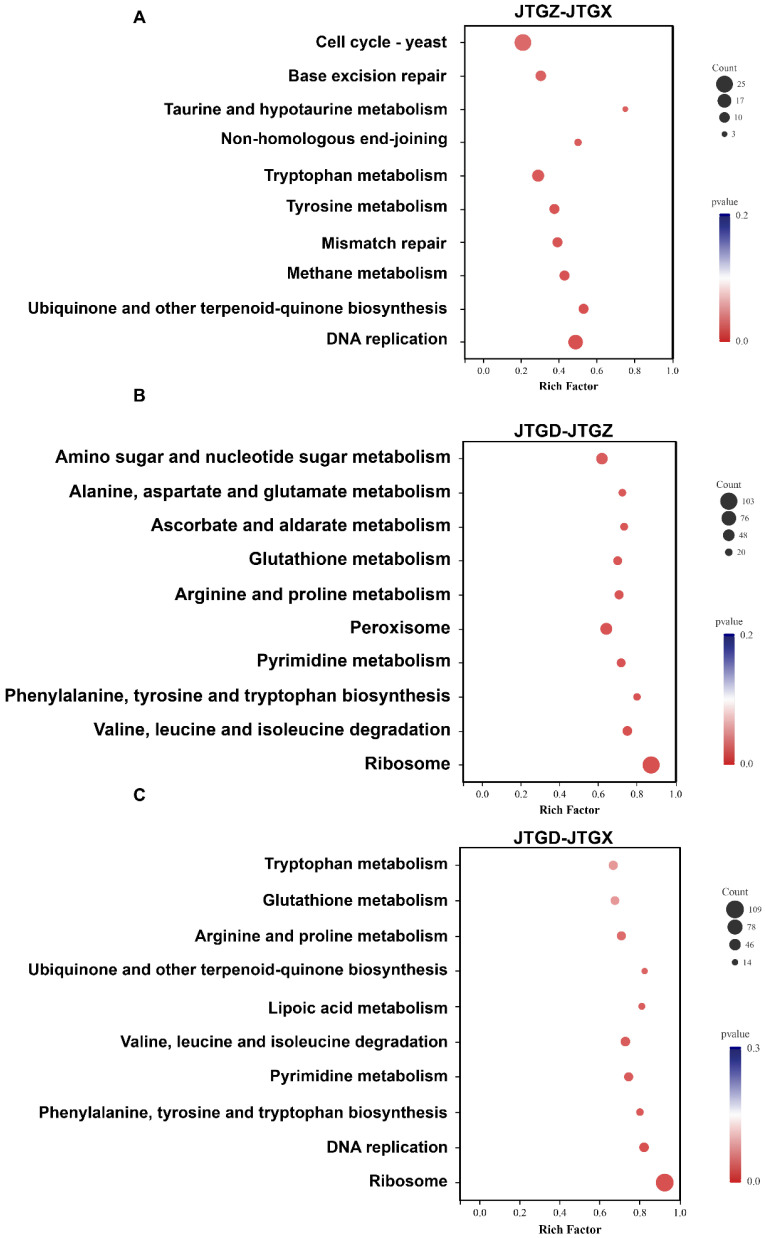
KEGG enrichment of differentially expressed genes across comparisons for *Coprinus comatus* development stages. DEGs (*p* < 0.05; |log_2_FC| ≥ 1) from each pairwise comparison were subjected to KEGG over-representation analysis. (**A**) JTGZ vs. JGTX Yeast cell cycle; base excision repair; taurine and hypotaurine metabolism; non-homologous end-joining; tryptophan metabolism; tyrosine metabolism; mismatch repair; methane metabolism; ubiquinone and other terpenoid–quinone biosynthesis; and DNA replication. (**B**) JTGD vs. JTGZ amino sugar and nucleotide sugar metabolism; alanine, aspartate, and glutamate metabolism; ascorbate and aldarate metabolism; glutathione metabolism; arginine and proline metabolism; peroxisome; pyrimidine metabolism; phenylalanine, tyrosine, and tryptophan biosynthesis; valine, leucine, and isoleucine degradation; and ribosome. (**C**) JTGD vs. JGTX tryptophan metabolism; glutathione metabolism; arginine and proline metabolism; ubiquinone and other terpenoid–quinone biosynthesis; lipoic acid metabolism; valine, leucine, and isoleucine degradation; pyrimidine metabolism; phenylalanine, tyrosine, and tryptophan biosynthesis; DNA replication; and ribosome.

KEGG enrichment of differentially expressed genes (*p* < 0.05; |log_2_FC| ≥ 1) corresponded to stage-specific developments: JTGZ vs. JGTX showed significant enrichment for the Yeast cell cycle, base excision repair, taurine and hypotaurine metabolism, non-homologous end-joining, tryptophan metabolism, tyrosine metabolism, mismatch repair, methane metabolism, ubiquinone and other terpenoid–quinone biosynthesis, and DNA replication (Figure 8A). JTGD vs. JTGZ was enriched for amino sugar and nucleotide sugar metabolism; alanine, aspartate, and glutamate metabolism; ascorbate and aldarate metabolism; glutathione metabolism; arginine and proline metabolism; peroxisome; pyrimidine metabolism; phenylalanine, tyrosine, and tryptophan biosynthesis; valine, leucine, and isoleucine degradation; and ribosome (Figure 8B). Finally, JTGD vs. JGTX was enriched for tryptophan metabolism; glutathione metabolism; arginine and proline metabolism; ubiquinone and other terpenoid–quinone biosynthesis; lipoic acid metabolism; valine, leucine, and isoleucine degradation; pyrimidine metabolism; phenylalanine, tyrosine, and tryptophan biosynthesis; DNA replication; and ribosome (Figure 8C). No pathway corresponded to all three contrasts. Pairwise overlaps included tryptophan metabolism, ubiquinone and other types of terpenoid–quinone biosynthesis, and DNA replication between JTGZ–JGTX and JTGD–JGTX. Also included were glutathione metabolism; arginine and proline metabolism; valine, leucine, and isoleucine degradation; pyrimidine metabolism; phenylalanine, tyrosine, and tryptophan biosynthesis; and ribosome between JTGD–JTGZ and JTGD–JGTX. All remaining terms were contrast-specific, as listed above (Figure 8A–C).

To further integrate transcriptomic and metabolomic variation across developmental stages, pairwise correlation analysis was performed between DEGs and DAMs for the three comparisons (JTGD–JTGX, JTGD–JTGZ, and JTGZ–JTGX). The correlation heatmaps (Supplemental Figure S1A–C) showed clear transcript–metabolite association patterns. Distinct correlation blocks were formed, where groups of DEGs displayed coordinated positive or negative correlations with multiple stage-responsive metabolites. Hierarchical clustering further revealed that DEGs with similar expression behaviors tended to correlate with similar metabolite sets, indicating the presence of tightly coordinated transcriptional–metabolic modules. These results further emphasize the close coupling between transcriptional regulation and metabolite accumulation during *C. comatus* development.

To further confirm the reliability of the RNA-seq data, six GST-encoding DEGs (10,361_g, 13,549_g, 12,316_g, 2176_g, 6680_g, and 12,317_g) from the glutathione metabolism pathway were selected for qRT-PCR validation. The qRT-PCR results (Supplementary Figure S2A–F) showed expression patterns highly consistent with the RNA-seq profiles across the three developmental stages, reinforcing the robustness of our DEG identification and supporting the involvement of GST-mediated redox regulation during *C. comatus* development.

## 4. Discussion

In this integrated transcriptomic–metabolomic analysis of *C. comatus*, we observed clear stage-dependent reprogramming of gene expression and metabolism as the fruiting bodies developed from the juvenile to mature stages. The monotonic trends evident in our data—with distinct clusters of genes and metabolites that either steadily increased or declined across the JTGX (juvenile), JTGZ (intermediate), and JTGD (mature) stages—underscore the orchestrated nature of mushroom development. Below, we discuss the biological significance of these trends in the context of mushroom physiology and how they align with or expand upon findings from other fungi.

Metabolites that decreased monotonically from juvenile to mature stages were largely associated with primary storage and structural compounds, whereas those that increased were often related to degradation products and secondary metabolism. For instance, we observed a pronounced depletion of various lipids (including triglycerides and phospholipids) as the mushroom matured. Juvenile fruiting bodies contained relatively high levels of membrane lipids and energy-rich fatty acids, which dropped steadily in intermediate and mature samples. This trend suggests that *C. comatus* mobilizes lipid reserves during development—likely consuming them for energy and membrane biosynthesis when developing the fruiting body, and/or reallocating them into spores as they form. It is well known that basidiospores accumulate substantial lipid bodies to fuel future germination [24]. In fact, ultrastructural studies show that during spore maturation, lipids are actively sequestered into the spores, and most mature mushroom spores are highly enriched in fats and sterols [24,25]. Therefore, the decline in free lipids in *C. comatus* fruiting tissue by the final development stage can be explained by the export of these lipids into spores and catabolic utilization. This observation is corroborated by gene expression data: we identified increased transcription of β-oxidation and peroxisome-related genes during the transition to maturity, which indicates active fatty acid breakdown and likely acts as an energy source during spore development and autolysis. Similar metabolic remodeling of lipids has been reported in other mushrooms; for example, during formation of primordium in *Pleurotus*, dramatic changes in lipid metabolism genes accompany the use of stored lipids to fuel morphogenesis [26].

In contrast to lipids, several nitrogenous metabolites accumulated in mature fruiting bodies. Notably, we detected a suite of small peptides (dipeptides and tripeptides) and amino-acid derivatives that were low in juveniles but rose significantly by the intermediate and especially the mature stage. Examples include dipeptides composed of hydrophobic amino acids (e.g., Leu-Leu and Ile-Phe) and compounds such as hydroxybutyrylcarnitine. The surge in these small peptides is a strong indicator that proteolysis and autolytic breakdown have commenced in the later stages of development. As the mushroom enters senescence, endogenous proteases likely degrade larger proteins into peptides and amino acids, either to recycle nutrients into spores or as a result of cellular self-digestion [1]. Autolysis in fungi involves broad hydrolysis of cellular constituents, as well as cell walls, cytoplasmic proteins, and organelles, which are broken down, releasing their contents [1]. The free peptides observed can, thus, be interpreted as hallmarks of incipient autolysis. This chemically substantiates the transcriptomic evidence of upregulated hydrolases. The accumulation of amino-acid derivatives like 3-hydroxybutyrylcarnitine in mature fruiting bodies may reflect metabolic stress responses (e.g., changes in mitochondrial fatty acid oxidation) or the repurposing of amino acids into energy substrates and protective compounds during the mushroom’s final stage. Interestingly, from a food science perspective, the increase in free amino acids and peptides in the later stages also contributes to the mushroom’s flavor and nutritional quality. Previous studies on edible fungi have shown that free amino-acid levels increase during fruiting body maturation, enhancing umami and overall taste in fully grown mushrooms [27]. In our case, the higher abundance of peptides found in mature *C. comatus* meant that older fruiting bodies have a richer savory flavor, though they are also on the verge of autodigestion. This trade-off between flavor (or nutrient availability) and structural integrity is an intriguing aspect of *C. comatus* biology that merits further study.

One key insight from our study is that many valuable compounds accumulate predominantly at the mature stage. Our findings align with those from a recent multi-omics analysis of another medicinal mushroom, *Lyophyllum decastes*, which demonstrated that total free amino acids and polysaccharides increased continuously through development and peaked in mature fruiting bodies [28]. Although we did not measure polysaccharide content for *C. comatus* directly, the transcriptomic upregulation of genes involved in cell-wall β-glucan synthesis and the general growth of structural polysaccharides suggest that polysaccharide levels (e.g., chitin, β-glucans) would be highest in fully developed fruiting bodies. These polysaccharides are largely responsible for the mushroom’s immunomodulatory and antioxidant activities [1]. They enable *C. comatus* to reach or approach full maturity, which could be used to maximize the yield of these bioactive polysaccharides and proteins in the harvested product. Likewise, certain secondary metabolites or nutraceuticals might only be synthesized in appreciable quantities at later stages. For example, if *C. comatus* produces any terpenoid compounds or unique secondary metabolites, this could indicate that relevant biosynthetic genes are more active in the mature stage (as demonstrated by enrichment of secondary metabolism pathways in JTGD). Harvesting too early might miss the period when these compounds accumulate.

The recurrence of glycerophospholipid metabolism across all contrasts indicates the persistent requirement for membrane–lipid remodeling throughout the development of *C. comatus* fruiting bodies. This is consistent with reports that phospholipid and broader lipid networks are reorganized during initiation and maturation in multiple basidiomycetes [29]. Pairwise overlaps reveal a bias for various compounds at different stages: biosynthesis of amino acids distinguishes the juvenile stage from both later stages, which is in line with high translational and precursor demands during early growth (as reflected by aminoacyl-tRNA/arginine/ABC-transporter terms in the juvenile→mid-stage comparison). Tyrosine metabolism marks transitions involving the mature stage, and is concordant with the late-stage reconfiguration of aromatic-amino-acid branches that feed diverse secondary metabolites [30,31,32]. Stage-specific (non-overlapping) pathways confirm this interpretation: the juvenile→mid-stage shift uniquely features aminoacyl-tRNA biosynthesis, arginine biosynthesis, ABC transporters, and porphyrin (heme) metabolism, consistent with intensified protein synthesis, nitrogen routing, transport capacity, and cofactor (heme) provisioning during early biogenesis [30,33]. D-amino-acid and cyanoamino-acid pathways, also specific to the juvenile→mid-stage contrast, map ancillary amino-acid derivatives (including cyanide-bearing species) and have been cataloged in KEGG as “other amino-acid metabolism.” They warrant cautious interpretation in fungi without targeted validation. By contrast, the mid-stage→mature step uniquely enriches pentose phosphate and TCA pathways, which is consistent with elevated NADPH supply and respiratory flux during maturation. This is observed alongside alpha-linolenic-acid metabolism, which is connected to oxylipin signaling implicated in fungal development [34,35]. Finally, tryptophan metabolism appears only in the endpoint contrast, reinforcing the late involvement of the aromatic network. By contrast, carbapenem biosynthesis should be viewed as a KEGG mapping label; it identifies shared intermediates rather than evidence of β-lactam production. Best-practice guidance warns that mis-annotation of metabolites can cause “antibiotic biosynthesis” in non-canonical organisms and advocates compound-level confirmation when drawing pathway-level conclusions [36].

The enrichment of DNA replication, cell-cycle, and multiple DNA repair modules in the juvenile→mid-stage comparison supports a proliferative/replicative program early in *Coprinus comatus* fruiting body development, aligning with basidiomycete transcriptomes that report stage-resolved upregulation of replication and cell-cycle machinery during early morphogenesis [37]. In contrast, the mid-stage→mature transition is dominated by amino-sugar/nucleotide-sugar metabolism (precursors for chitin/β-glucan and glycosylation), consistent with enhanced cell-wall biogenesis and remodeling. This interpretation is supported by foundational and recent work linking central sugar/nucleotide-sugar metabolism to fungal wall assembly [38,39,40]. Concomitant enrichment of glutathione metabolism and peroxisome indicates intensified redox buffering and β-oxidation-linked lipid turnover in maturation, which are well-documented requirements for fungal development and sexual reproduction [41,42,43]. Pairwise overlaps that involve the mature stage—including ribosome, pyrimidine metabolism, branched-chain amino-acid (BCAA) degradation, arginine/proline, and aromatic amino-acid biosynthesis—point to coordinated translational capacity, nucleotide supply, and amino-acid turnover during tissue differentiation and sporulation. This is in agreement with omics studies of mushroom-forming fungi and the energetic role of amino-acid catabolism under specific growth regimes [44,45,46,47]. Endpoint-specific cofactors—such as lipoic acid metabolism and ubiquinone/other terpenoid–quinone biosynthesis—further implicate respiratory-chain function and lipoylation-dependent 2-oxoacid dehydrogenase complexes as indicators of maturation [48,49]. Finally, taurine/hypotaurine and methane metabolism appear only in the juvenile→mid-stage set; because these KEGG maps can be triggered by promiscuous enzyme annotations or shared one-carbon/organosulfur intermediates outside canonical taxa, they warrant cautious interpretation and, ideally, compound-level validation before assigning mechanistic roles in basidiomycetes [50].

This integrative transcriptomic–metabolomic analysis revealed that *Coprinus comatus* undergoes highly coordinated and monotonic molecular reprogramming during fruiting body development, characterized by lipid depletion, accumulation of peptides and amino acids, and stage-specific enrichment of cell cycle, amino-acid, glutathione, and cell-wall metabolism pathways. These findings not only provide mechanistic insights into maturation and autolysis but also underscore the fact that bioactive metabolites peak at later stages, offering practical guidance for optimizing harvest times and cultivation strategies to enhance both yield and nutraceutical value.

## Data Availability

Data are deposited in the National Microbiology Data Center (NMDC) with accession number NMDC10020085 (https://nmdc.cn/resource/genomics/project/detail/NMDC10020085 accessed on 23 September 2025).

## Supplementary Materials

The following supporting information can be downloaded at https://www.mdpi.com/article/10.3390/jof11120849/s1, Figure S1: Correlation analysis between DEGs and DAMs in the three developmental stage comparisons; Figure S2: qRT-PCR validation of six GST-encoding DEGs across the three developmental stages of Coprinus comatus; Table S1: The sequences of the primers used in this study.

## Abbreviations

The following abbreviations are used in this manuscript:

ABC	ATP-binding cassette transporters
BCA	branched-chain amino acids
cDNA	complementary DNA
DAMs	differentially abundant metabolites
DEGs	differentially expressed genes
GO	Gene Ontology
HMDB	Human Metabolome Database
KEGG	Kyoto Encyclopedia of Genes and Genomes
LC–MS	liquid chromatography–mass spectrometry
mRNA	messenger RNA
OPLS–DA	orthogonal partial least squares–discriminant analysis
PCA	principal component analysis
PCR	polymerase chain reaction
QC	quality control
RNA	ribonucleic acid
RNA-seq	RNA sequencing
TCA	tricarboxylic acid cycle
TPM	transcripts per million
VIP	variable importance in projection
