# Supplementary material for: Integrated Transcriptomic and Metabolomic Profiling Reveals Monotonic Molecular Signatures During Fruiting Body Development of Coprinus comatus
Source: J Fungi (Basel). 2025 Nov 29;11(12):849. doi: 10.3390/jof11120849 (PMC12733512; doi:10.3390/jof11120849)
Supplement: Supplementary file 1 [file jof-11-00849-s001.zip › Supplementary figure.pdf]

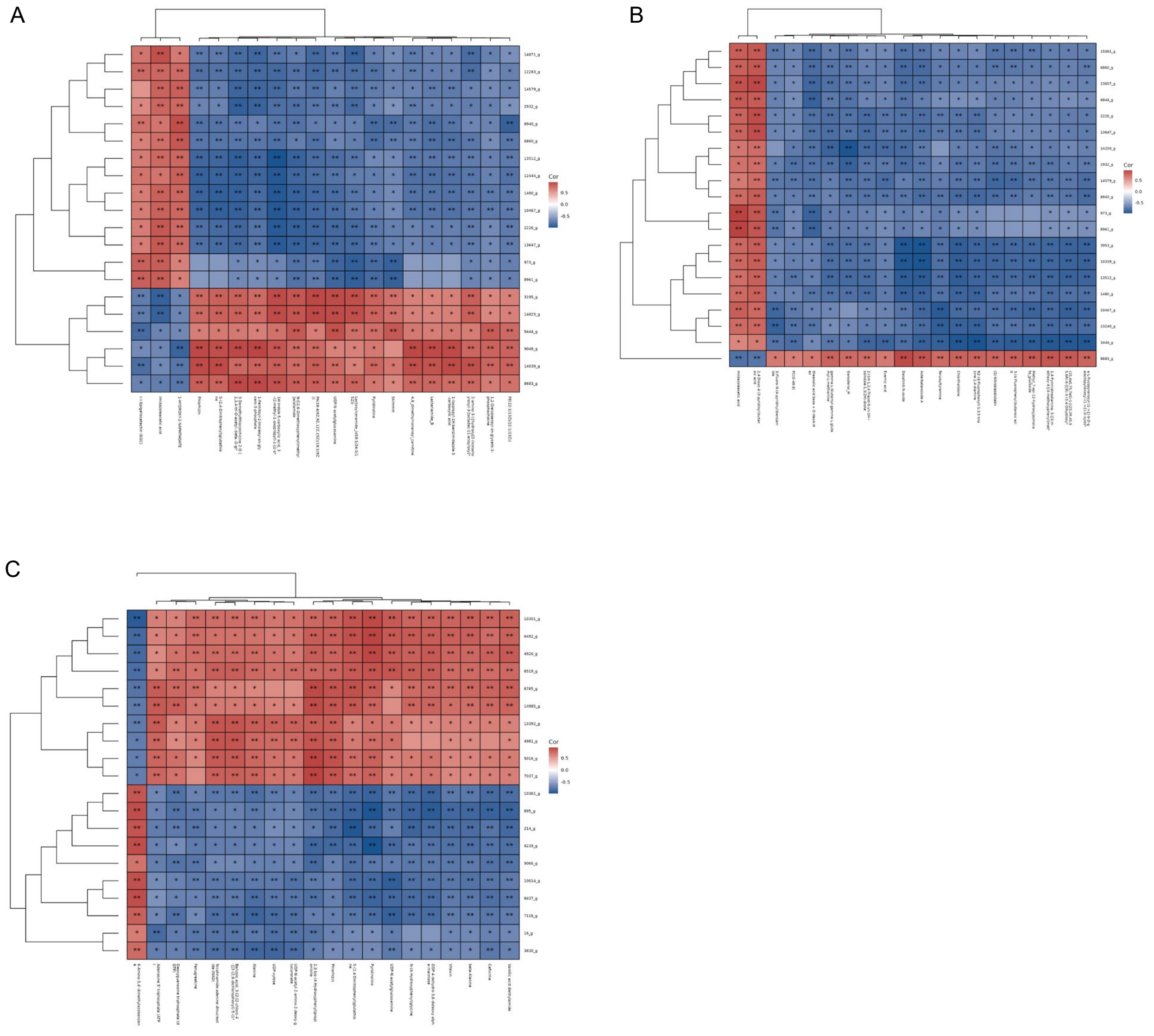

Supplemental Figure S1. Correlation analysis between DEGs and DAMs in the three developmental stage comparisons.

(A) JTGD vs. JTGX; (B) JTGD vs. JTGZ; (C) JTGZ vs. JTGX. Heatmaps display Pearson correlation coefficients between differentially expressed genes (right axis) and differentially accumulated metabolites (bottom axis). The left and top dendrograms represent hierarchical clustering based on correlation similarity; closer branches indicate genes or metabolites with more similar correlation patterns. Color intensity reflects correlation strength, with red representing positive correlations and blue representing negative correlations. Values closer to ±1 indicate stronger correlations. Asterisks denote significance levels of the correlation test: $p \leq 0.05$ (*) and $p \leq 0.01$ (**).

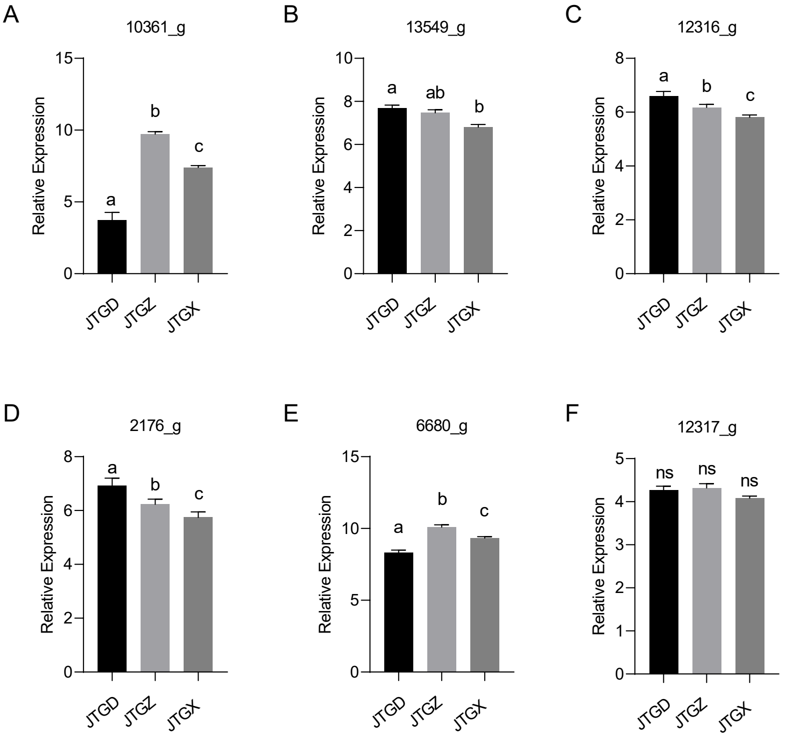

Supplementary figure S2. qRT-PCR validation of six GST-encoding DEGs across the three developmental stages of Coprinus comatus. Relative expression levels of six glutathione S-transferase (GST) genes (*10361_g*, *13549_g*, *12316_g*, *2176_g*, *6680_g*, and *12317_g*) were quantified in JTGX, JTGZ, and JTGD stages. Bars represent mean ± SD of three biological replicates. Different lowercase letters indicate a significant difference at $P < 0.05$ based on one-way analysis of variance (ANOVA).
